# Sensitivity is not an intrinsic property of a diagnostic test: empirical evidence from histological diagnosis of *Helicobacter pylori *infection

**DOI:** 10.1186/1471-230X-9-98

**Published:** 2009-12-24

**Authors:** Nuno Lunet, Bárbara Peleteiro, Carla Carrilho, Céu Figueiredo, Ana Azevedo

**Affiliations:** 1 Department of Hygiene and Epidemiology, University of Porto Medical School, Porto, Portugal; 2 Institute of Public Health - University of Porto (ISPUP), Porto, Portugal; 3 Department of Pathology, Medical Faculty, Eduardo Mondlane University, Maputo, Mozambique; 4 Institute of Molecular Pathology and Immunology of the University of Porto (IPATIMUP), Porto, Portugal; 5 Department of Pathology, University of Porto Medical School, Porto, Portugal

## Abstract

**Background:**

We aimed to provide empirical evidence of how spectrum effects can affect the sensitivity of histological assessment of *Helicobacter pylori *infection, which may contribute to explain the heterogeneity in prevalence estimates across populations with expectedly similar prevalence.

**Methods:**

Cross-sectional evaluation of dyspeptic subjects undergoing upper digestive endoscopy, including collection of biopsy specimens from the greater curvature of the antrum for assessment of *H. pylori *infection by histopathological study and polymerase chain reaction (PCR), from Portugal (n = 106) and Mozambique (n = 102) following the same standardized protocol.

**Results:**

In the Portuguese sample the prevalence of infection was 95.3% by histological assessment and 98.1% by PCR. In the Mozambican sample the prevalence was 63.7% and 93.1%, respectively. Among those classified as infected by PCR, the sensitivity of histological assessment was 96.2% among the Portuguese and 66.3% among the Mozambican. Among those testing positive by both methods, 5.0% of the Portuguese and 20.6% of the Mozambican had mild density of colonization.

**Conclusions:**

This study shows a lower sensitivity of histological assessment of *H. pylori *infection in Mozambican dyspeptic patients compared to the Portuguese, which may be explained by differences in the density of colonization, and may contribute to explain the heterogeneity in prevalence estimates across African settings.

## Background

The dependence of positive and negative predictive values of diagnostic tests on the frequency of the target condition in the population under study is widely recognized and easy to quantify, as predictive values are seen as post-test probabilities of disease, determined by the pre-test probability which is changed by knowledge of the results of the test. Sensitivity and specificity, despite being stable across the settings in which the test is used, actually also depend on the characteristics of patients in whom those parameters were assessed. There is much evidence for varying specificity, depending on alternative diagnoses and comorbid conditions that affect patients without the target disease and which may affect the test. Sensitivity is conditioned by the severity of the disease that is being diagnosed and comorbid conditions that may lead to false negative results [1]. However, this possibility is often not acknowledged, since these situations are much less frequently reported [2].

The external validity of the results of studies evaluating the accuracy of a diagnostic test may be affected by variations in case mix - spectrum effects - either as a consequence of flawed study designs [3], or resulting from true variation across populations. Examples of the latter, however, are seldom described.

We provide empirical evidence of how spectrum effects may affect the sensitivity of histological assessment of *Helicobacter pylori *infection, in two different settings, Portugal and Mozambique, in which high prevalences of infection in the general population are to be expected [4].

## Methods

Dyspeptic subjects from Portugal and Mozambique underwent upper endoscopy evaluation and gastric biopsy samples were collected following the same standardized protocol, including the collection of biopsy specimens from the greater curvature of the antrum for assessment of *H. pylori *infection by histopathological study and polymerase chain reaction (PCR), as previously described [5,6].

The ethics committees of involved institutions approved the study, and all participants provided written informed consent.

### Portuguese sample [5]

All workers from the Viana do Castelo shipyard, north of Portugal, were invited for a gastric pathology survey in 1998. Four hundred and sixty volunteered to the study and completed a physician-administered questionnaire on digestive symptoms. An upper digestive endoscopy was performed to 107 participants reporting dyspeptic symptoms and biopsy samples from the antrum were available from 106 participants (median age: 44 years, interquartile range: 39-48 years; 96.2% men; 100% white).

### Mozambican sample [6]

Among patients observed at the outpatient department of Gastroenterology at the Maputo Central Hospital, adults with dyspeptic symptoms and clinical criteria for upper digestive endoscopy were consecutively invited to this study between August 2005 and May 2006. One gastric cancer patient was excluded and the present series includes 102 participants for whom antrum biopsies with adequate size were considered for histopathological study (median age: 37 years, interquartile range: 29-46 years; 34.3% men; 93.1% black).

### Collection of biopsy specimens

The protocol included the collection of four biopsy specimens--one from the greater curvature of the antrum, two from the *incisura angularis*, and one from the anterior wall of the corpus, for the histopathological study. In the Mozambican sample only 57 subjects had biopsies from the corpus available due to a high frequency of erroneous labelling as corpus of samples collected from antrum or *incisura*.

In the Portuguese participants an additional antral biopsy specimen was collected for PCR analysis and for the Mozambican subjects an antral biopsy used for histopathological study was also was used for PCR.

### Histopathological study

Biopsy specimens were fixed in 10% formalin and embedded in paraffin and cut into 4-μm sections. Modified Giemsa stained sections were used to identify *H. pylori*. Histological evaluation of samples from both settings was performed according to the Modified Sydney system [7] by two experienced pathologists, using a semi-quantitative scoring (0 = absent; 1 = mild; 2 = moderate; 3 = marked) for density of colonization with *H. pylori*.

### Assessment of *H. pylori *infection by PCR

In Portuguese patients, total DNA was extracted from frozen antral biopsy specimens using the method described by Boom *et al *[8]. Briefly, biopsies were homogenized in guanidinium isothiocyanate, using a sterile micropestle. DNA was captured onto silica particles, washed, and eluted in 100 ml of 10 mmol/L Tris-HCl, pH 8.3. Two microliters were used for each PCR. *H. pylori *detection was performed by multiplex amplification of *vacA *and *cagA *genes followed by reverse hybridization on a line probe assay (LiPA) as described earlier [9].

In Mozambican samples, DNA was extracted from formalin-fixed, paraffin-embedded antral biopsy specimens after digestion with Proteinase K for at least 12 hours at 55°C. Proteinase K was inactivated by incubation at 95°C for 10 minutes. Ten microliters of the lysate were used for PCR. Detection of *H. pylori *was performed with the same primers used for the Portuguese samples. Amplified products were visualised after electrophoresis in 2% agarose gels.

### Data analysis

Since only one biopsy (from the antrum) was used for PCR, we compared the results of PCR assessment of infection with those from histological assessment using a biopsy collected from a similar location, to ensure the comparability of the two results obtained with the two methods. PCR was defined as the reference standard and sensitivity of histological assessment was computed with respective 95% Confidence Intervals (95%CI). Proportions were compared using the χ ^2 ^test.

Sensitivity analyses were also conducted comparing the results from the histological assessment of infection in four biopsies collected from different stomach locations with those from PCR evaluation of the antral specimen, and restricting the analysis to subjects not reporting previous eradication and/or proton pump inhibitors (PPI)/H2 receptor blockers treatment.

## Results

In the Portuguese sample the prevalence of infection was 95.3% by histological assessment and 98.1% by PCR. In the Mozambican sample the prevalence was 63.7% and 93.1%, respectively.

Among those classified as infected by PCR, 96.2% of the Portuguese and 66.3% of the Mozambican participants yielded a positive result by histological assessment. The sensitivity estimates were similar when only the subjects reporting no previous eradication and/or PPI/H2 receptor blockers treatment were considered (Table 1). Among those testing positive by both methods, 5.0% of the Portuguese and 20.6% of the Mozambican had mild density of colonization (χ ^2 ^test, 2 degrees of freedom: p = 0.008).

**Table 1:** Sensitivity of histological assessment of *Helicobacter pylori *infection using PCR analysis as the reference standard, in Portuguese and Mozambican populations of dyspeptic patients.

	*Helicobacter pylori *infection			
	
	PCR (reference standard)	Histological assessment (index text)		
		
	+	+	-	Sensitivity (95%CI)
All participants				
Portugal	104	100*	4	96.2 (90.4-98.9)
Mozambique	95	63†	32	66.3 (55.9-75.7)
Participants not reporting previous eradication treatment				
Portugal	97	95‡	2	97.9 (92.7-99.7)
Mozambique	62	41§	21	66.1 (53.0-77.7)
Participants not reporting previous PPI/H2 receptor blockers treatment				
Portugal	75	74#	1	98.7 (92.8-100.0)
Mozambique	37	22¶	15	59.4 (42.1-75.2)
Excluding participants reporting previous eradication and PPI/H2 receptor blockers treatments				
Portugal	97	95**	2	97.9 (92.7-99.7)
Mozambique	66	42††	24	63.6 (50.9-75.1)

PCR - Polymerase chain reaction; 95%CI - 95% confidence interval; PPI - Proton pump inhibitors * *H. pylori *density score: mild (5.0%); moderate (42.0%); marked (53.0%), † *H. pylori *density score: mild (20.6%); moderate (34.9%); marked (44.4%), ‡ *H. pylori *density score: mild (5.3%); moderate (42.1%); marked (52.6%), §*H. pylori *density score: mild (19.5%); moderate (43.9%); marked (36.6%), # *H. pylori *density score: mild (5.4%); moderate (44.6%); marked (50.0%), ¶*H. pylori *density score: mild (27.3%); moderate (40.9%); marked (31.8%), ** *H. pylori *density score: mild (5.3%); moderate (42.1%); marked (52.6%), †† *H. pylori *density score: mild (21.4%); moderate (42.9%); marked (35.7%)

Among the Mozambicans, sensitivity was not improved when defining infection by histopathological analysis of 4 biopsy samples from corpus, antrum and *incisura angularis *(66.7%, 95%CI: 52.9%-78.5%).

Thirty percent of the Mozambican subjects reported a previous eradication treatment, with a similar prevalence of infection and *H. pylori *density score regardless of treatment (previous eradication therapy *vs*. no previous treatment: no infection - 34.5% *vs*. 33.9%; mild - 17.2% *vs*. 12.9%; moderate - 10.3% *vs*. 29.0%; marked - 37.9% *vs*. 24.2%, p = 0.195). Fifty-seven percent of the Mozambican subjects reported a previous treatment with PPI/H2 receptor blockers with a similar prevalence of infection and *H. pylori *density score regardless of treatment (previous treatment with PPI/H2 receptor blockers *vs*. no previous treatment: no infection - 29.6% *vs*. 40.5%; mild - 11.1% *vs*. 16.2%; moderate - 22.2% *vs*. 24.3%; marked - 37.0% *vs*. 18.9%, p = 0.304). Among the Portuguese participants, 6% reported previous eradication therapy, and 25% declared to have used PPI/H2 receptor blockers.

## Discussion

The sensitivity of the histological assessment of *H. pylori *infection was considerably lower in Mozambique than in Portugal, which may be explained by a lower density of colonization among Mozambican patients.

In Portuguese samples, DNA was extracted from frozen antral biopsies and used for PCR, followed by reverse hybridization onto specific probes, whereas in Mozambican samples, DNA was extracted from formalin-fixed, paraffin-embedded biopsies and used for PCR, followed by electrophoresis in agarose gels stained with ethidium bromide. Visual inspection of PCR products on agarose gels provides only limited reliability, as compared to hybridization to allele-specific probes. However, it has been previously shown that *H. pylori *genotypes obtained from paraffin sections matched those corresponding cultured strains [10]. Nevertheless, even if PCR results for Mozambican samples were underestimated, this would mean that the sensitivity of histological assessment for this population would even be lower, and more discrepant than those obtained for the Portuguese samples.

Co-infections with other *Helicobacter *species have been described in African populations [11], but the PCR analysis that we have used is specific for *H. pylori *(we have used primers directed to the *vacA *gene which is only present in this species). Primer sequences used in this study were analyzed for specificity by using the Blast program at the National Institute of Health Data libraries [12].

The histological identification of *H. pylori *in tissue sections may be affected by biopsy and observer-related factors. Sensitivity is higher when at least samples from the antrum and the body are analyzed, but the proportion of infected subjects did not vary meaningfully when we considered the histological assessment of infection status using biopsies from the three locations, which is in accordance with the fact that the antrum biopsies tend to yield a higher prevalence of infection than the other locations [13], strengthening the validity of our conclusions. Moreover, no meaningful increase in the prevalence of infection when assessed by PCR was to be expected, as it was well above 90% when only one biopsy is used for this purpose.

Staining methods such as modified Giemsa are highly sensitive, but histological examination relies on the experience of the histopathologist to recognize the typical morphology of the organism, and small numbers of organisms may be missed [14,15], while PCR methods have been described as more sensitive and not dependent on the level of experience of the pathologist [16,17].

In our study, samples from both settings were collected and processed following similar methods and were evaluated by the same experienced pathologists. It is unlikely that differences in technical aspects or inter-observer and laboratory variations explain the differences in the results obtained in Portuguese and Mozambican samples. The same applies to the grading of the density of colonization, which was conducted following the same criteria in both samples.

Although coccoid forms (suggesting possible degenerative forms of *H. pylori*) could have been found in the negative specimens, and would contribute to the low perceived sensitivity of histological assessment of infection, the pathologists where aware of this possibility. Even considering the difficulties in classifying correctly these uncharacteristic forms in the absence of findings of structures with the characteristic *H. pylori *shape, these were seldom observed and the differences observed in sensitivity or in the density of *H. pylori *colonization cannot be ascribed to such phenomenon.

A lower sensitivity of histology for the assessment of *H. pylori *infection has been recognized after partially effective eradication therapy [15,16], as low levels of recurrent infection can be easily missed by biopsy, leading to overestimation of therapeutic efficacy [18]. The proportion of Mozambican subjects reporting a previous eradication treatment was surprisingly high, which could explain the lower density of colonization among these patients, but no information was obtained regarding the success of eradication or time since eradication. No significant differences were found in infection status or density of *H. pylori *colonization according to self-reported eradication treatment, probably reflecting the large potential for erroneous reporting of such a specific therapy, and sensitivity was similar when only subjects not reporting an eradication treatment were considered. Similarly, the previous use of PPI/H2 receptor blockers was also self-reported and no information was collected regarding current treatment or time since last treatment course, but sensitivity was similar when only subjects not reporting the use of PPI/H2 receptor blockers were considered.

Gastric atrophy and intestinal metaplasia lead to a pH increase in the stomach, which can create an unfavorable environment for *H. pylori *survival, and could contribute to lower density of colonization [19,20]. The density of *H. pylori *may be increased in macroscopic erosions [21]. However, only 14.7% of the Mozambican patients had chronic atrophic gastritis (5.9%) or intestinal metaplasia (8.8%), in comparison with 43.9% in the Portuguese subjects (chronic atrophic gastritis: 6.5%; intestinal metaplasia: 37.4%), and erosions/ulcerations were observed in one Portuguese and four Mozambicans. Therefore, the unequal distribution of these lesions could not explain the differences in the prevalence of infection detected by histology.

Although the samples were evaluated in different time periods and present different characteristics regarding the participants' ethnicity and gender, our analysis only included subjects testing positive for *H. pylori *infection by PCR and the above differences between the Portuguese and the Mozambican sample are not likely to account for our conclusions.

In studies conducted in dyspeptic patients from different African populations the overall prevalence of *H. pylori *infection, assessed by histology, was approximately 72%, varying from 25% in Uganda to 97% in Ghana [22], although a high prevalence of infection is expected given its strong association with low socioeconomic status. The heterogeneity of estimates across African settings, as well as unexpectedly low figures, is likely to be explained both by observer-related factors and differences in the density of colonization by *H. pylori*.

Mozambique and other African countries have a low frequency of gastric cancer and gastric precancerous lesions, despite the high prevalence of infection [23,24], reflecting what has been called the African enigma. In addition to providing an example of how spectrum effects can affect the sensitivity of histological assessment of *H. pylori *infection, our results add a piece of information to our current understanding of the "enigma", suggesting that despite the absence of differences in the frequency of infection with the more virulent strains in Portugal and Mozambique [25], the higher prevalence of mild infections in Africa may be associated with a lower incidence of cancer.

## Conclusions

This study provides empirical evidence of how subtle spectrum effects, not induced by a flawed study design, may be responsible for large differences on the sensitivity of histological assessment of *H. pylori *infection, and may contribute to explain the heterogeneity in prevalence estimates across African settings.

## Competing interests

The authors declare that they have no competing interests.

## Authors' contributions

NL has made substantial contributions to conception and design, acquisition of data, and analysis and interpretation of data, as well as has been involved in drafting the manuscript. BP has made substantial contributions to conception and design, acquisition of data, and analysis and interpretation of data. CC has made substantial contributions to conception and design and acquisition of data. CF has made substantial contributions to conception and design and acquisition of data. AA has been involved in conception and design, drafting the manuscript and revising it critically for important intellectual content. All authors have given final approval of the version to be published.

## Pre-publication history

The pre-publication history for this paper can be accessed here:

http://www.biomedcentral.com/1471-230X/9/98/prepub
